# Make it STING: nanotechnological approaches for activating cGAS/STING as an immunomodulatory node in osteosarcoma

**DOI:** 10.3389/fimmu.2024.1403538

**Published:** 2024-09-30

**Authors:** Jordan C. O’Donoghue, Fiona E. Freeman

**Affiliations:** ^1^ School of Mechanical and Materials Engineering, Engineering and Materials Science Centre, University College Dublin, Dublin, Ireland; ^2^ University College Dublin (UCD) Centre for Biomedical Engineering, University College Dublin, Belfield Dublin, Ireland; ^3^ Conway Institute of Biomolecular and Biomedical Research, University College Dublin, Dublin, Ireland; ^4^ Trinity Centre for Biomedical Engineering, Trinity Biomedical Sciences Institute, Trinity College Dublin, Dublin, Ireland; ^5^ Department of Mechanical Manufacturing, and Biomedical Engineering, School of Engineering, Trinity College Dublin, Dublin, Ireland; ^6^ Advanced Materials and Bioengineering Research Centre (AMBER), Royal College of Surgeons in Ireland and Trinity College Dublin, Dublin, Ireland; ^7^ I-Form Centre, School of Mechanical and Materials Engineering, University College Dublin (UCD), Dublin, Ireland

**Keywords:** osteosarcoma, STING, cGAS, immunotherapy, innate immunity, nanotechnology, drug delivery

## Abstract

Osteosarcoma is a highly aggressive bone cancer primarily affecting children, adolescents, and young adults. The current gold standard for treatment of osteosarcoma patients consists of two to three rounds of chemotherapy, followed by extensive surgical intervention from total limb reconstruction to amputation, followed by additional rounds of chemotherapy. Although chemotherapy has advanced the treatment of osteosarcoma significantly, the overall 5-year survival rate in resistant forms of osteosarcoma is still below 20%. The interaction between cancer and the immune system has long been recognized as a critical aspect of tumour growth. Tumour cells within the tumour microenvironment (TME) suppress antitumour immunity, and immunosuppressive cells and cytokines provide the extrinsic factors of tumour drug resistance. Emerging research demonstrates an immunostimulatory role for the cGAS/STING pathway in osteosarcoma, typically considered an immune-cold or immunosuppressed cancer type. cGAS/STING signalling appears to drive an innate immune response against tumours and potentiates the efficacy of other common therapies including chemo and radiotherapy. Nanotechnological delivery systems for improved therapy delivery for osteosarcoma have also been under investigation in recent years. This review provides an overview of cGAS/STING signalling, its divergent roles in the context of cancer, and collates current research which activates cGAS/STING as an adjuvant immunomodulatory target for the treatment of osteosarcoma. It will also discuss current nanotechnological delivery approaches that have been developed to stimulate cGAS/STING. Finally, it will highlight the future directions that we believe will be central to the development of this transformative field.

## 1 Introduction

Osteosarcoma is a highly aggressive bone cancer, largely affecting children, adolescents, and young adults (1–3). The current gold-standard treatment regimen for osteosarcoma involves neo-adjuvant chemotherapy followed by either resection of the affected region in conjunction with limb remodelling or complete limb amputation (4–6). Surgical intervention is then complemented by adjuvant chemotherapy to eliminate any circulating tumour cells. Common chemotherapeutics used in this process include anthracyclines (doxorubicin), alkylating agents (cisplatin or ifosfamide) and anti-metabolites (methotrexate) (3, 6). Typically, co-administration of two or more of these agents is required due to the aggressive nature of osteosarcoma. However, although chemotherapy has advanced the treatment of osteosarcoma significantly, the overall 5-year survival rate in resistant forms of osteosarcoma is still below 24% (7, 8). Critically, approximately 25-45% of osteosarcoma patients display chemoresistance and thus are prone to relapse and recurrence (9). Furthermore, chemoresistance and radioresistance has been attributed to various characteristics inherent to osteosarcoma tumours such as increased expression of drug efflux transporters, enhanced capacity for DNA damage repair, and a high degree of autophagic flux. The development of chemoresistance presents a major challenge in effectively treating osteosarcoma, and overcoming this challenge is crucial for better patient outcomes (10, 11). The interaction of cancer and the immune system has long been recognised as a critical modulator of chemoresistance. Tumour cells within the TME suppress antitumour immunity through the expression of immune checkpoint inhibitors. Furthermore, tumour associated macrophages and cancer associated fibroblasts secrete various proteins, including immunosuppressive cytokines, such as transforming growth factor-ß (TGF-ß), interleukin 10 (IL-10), indolamine-2,3-dixoygenase (IDO) and vascular endothelial growth factor (VEGF) (12, 13). These cells and proteins provide the extrinsic factors of tumour drug resistance. Various immunotherapies have been investigated for the management of osteosarcoma since osteosarcoma tumours frequently express high levels of key immune checkpoint inhibitor proteins (namely programmed cell death protein 1 (PD-1), programmed death-ligand 1 (PD-L1), Cytotoxic T-lymphocyte associated protein 4 (CTLA4), and T-cell immunoglobulin and mucin domain 3 (TIM3)) (14–17). Unfortunately, the implementation of immunotherapies alone has failed to provide significant clinical benefit to patients with more aggressive and metastatic cases of osteosarcoma.

Chemoimmunotherapy combinations have attracted increasing attention as one of the most effective synergistic strategies against aggressive tumours like osteosarcoma. In addition to playing their respective roles, chemotherapy and immunotherapy can synergistically promote each other’s immunogenic effects. Specifically, the cyclic GMP-AMP synthase/Stimulator of Interferon Genes (cGAS/STING) pathway is an emerging immunomodulatory node due to its role in type 1 interferon production and the upregulation of antigen presentation, dendritic cell maturation, and CD8+ T-lymphocyte-mediated tumour cell clearance (18–20). cGAS/STING activation, in the context of cancer, occurs following detection of tumour derived micronuclei and nuclear double stranded DNA (dsDNA) fragments. Reports indicate cGAS/STING dysregulation at various levels in a variety of cancers, including osteosarcoma, elucidated using *in vitro* models (21–23). Gene silencing of pathway components, dysfunctional post-translational modification of pathway components, and increased degradation of cGAS ligands are observed in numerous cancers and correlate with a more malignant phenotype (21). This review provides an overview of the cGAS/STING pathway, its divergent roles in tumour elimination and tumorigenesis, and reported approaches to activating cGAS/STING signalling as a means of driving tumour elimination. We will discuss the rational and challenges involved in the targeting of cGAS/STING as an adjuvant target alongside the administration of chemo/radiotherapy. Finally, we will highlight some future directions that we believe will be central to the development of this transformative field.

## 2 cGAS/STING pathway

**Figure 1:**
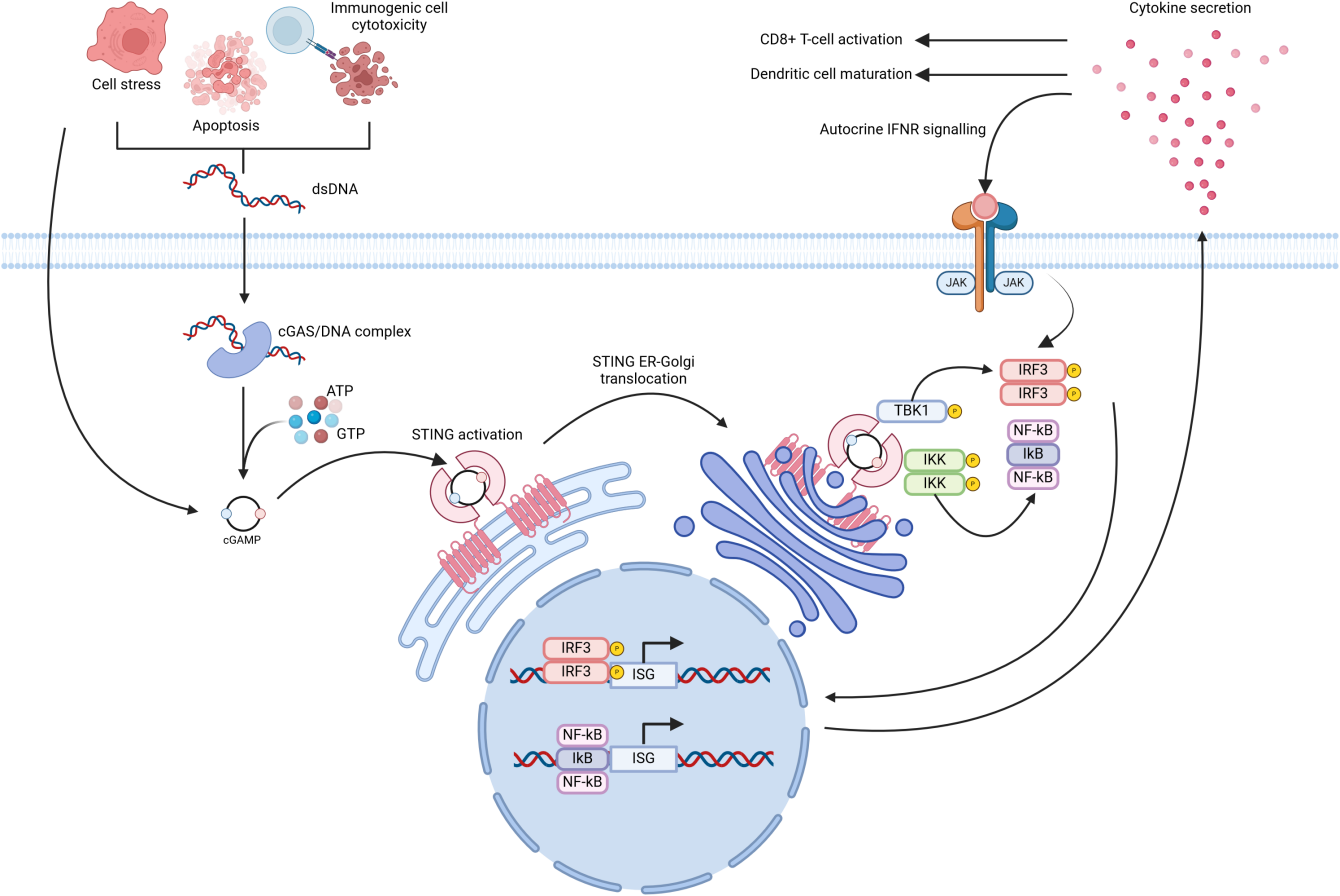
Diagrammatic overview of cGAS/STING signal transduction. Release of double stranded DNA (dsDNA) from cells, due to apoptosis or cellular stress, leads to cGAS homodimerization and subsequent activation of its catalytic activity. cGAS utilises cytosolic ATP and GTP as substrates for the formation of cGAMP. Cells may also internalise cGAMP present in the extracellular milieu which is released in a similar fashion to that of dsNDA. Inactive STING, located at the endoplasmic reticulum, binds cGAMP and undergoes translocation to the Golgi apparatus wherein it recruits Tank-binding kinase 1 (TBK1). Active TBK1 in-turn phosphorylates Interferon regulatory factor 3 (IRF3) and IkB kinase (IKK). These events culminate in the expression of numerous Interferon stimulated genes (ISGs) through the synergistic activity of IRF3 and NF-kB. Created with BioRender.com.

The cGAS/STING pathway was initially described as a defence mechanism versus intracellular pathogens, namely bacteria and viruses. It is now understood that the cGAS/STING pathway coordinates a type 1 interferon (IFN-1) based immune response following detection of exogenous DNA (pathogen-derived) or self-DNA (released due to cellular stress and/or apoptosis) (24). cGAS/STING signalling begins with the detection of dsDNA by cGAS, which is located in the cytosol and belongs to the nucleotidyl transferase enzyme superfamily (24, 25). DNA binding to cGAS causes cGAS dimerization and subsequent activation of catalytic activity (26). Active cGAS synthesises 2’3’-cyclic guanosine-adenosine monophosphate (2’3’-cGAMP) using adenosine triphosphate and guanosine triphosphate as substrates. Next, 2’3’-cGAMP binds to and activates STING homodimers present in the endoplasmic reticulum (ER) (27, 28). 2’3’-cGAMP binding to STING induces a pronounced conformational change leading to STING translocation, through the ER-Golgi intermediate compartment, to the Golgi apparatus (25, 29) (**Figure 1**). STING translocation is believed to occur in a COPII complex dependent fashion (30, 31). Once situated in the Golgi, STING recruits TANK-binding kinase 1 (TBK1), mediated through a conserved PLPLRT/SD amino acid binding motif located within STING (32) (**Figure 1**). TBK1 recruitment facilitates TBK1 dimerisation and subsequent autophosphorylation. Active TBK1 promotes the recruitment of interferon-regulatory factor 3 (IRF3) to the STING/TBK1 complex through phosphorylation of STING on Ser366 (25, 30). Following recruitment, IRF3 undergoes phosphorylation by TBK1 thus inducing IRF3 dimerisation and nuclear translocation wherein its binds and activates the expression of interferon stimulated genes (ISGs) (**Figure 1**). STING has also been seen to facilitate IκB kinase (IKK) phosphorylation leading to the activation of the heterotrimeric IκB/Nuclear Factor-kappa B (NF-κB) complex and its translocation to the nucleus, wherein it collaborates with active IRF3 homodimers to drive a dual type 1 interferon/inflammatory cytokine transcriptional profile (24, 25, 30) (**Figure 1**). Synergistic activation of IRF3 and IκB/NF-κB transcriptional control leads to the expression of ISGs with diverse products such as type 1 interferons, inflammatory cytokines (IL-6, TNF-α), and chemokines (CCL5, CXCL10) (25, 33, 34). Type 1 interferons produced in this manner can act signal in an autocrine fashion to activate IFN receptors, culminating in Signal Transducer and Activator of Transcription (STAT) protein activation, through Janus Kinase (JAK) signalling. This feedback loop further amplifies the production of inflammatory and immune-stimulatory mediators (**Figure 1**).

## 3 Anti-tumour effects of cGAS/STING signalling:

### 3.1 Mechanisms of cGAS/STING activation in the TME

**Figure 2:**
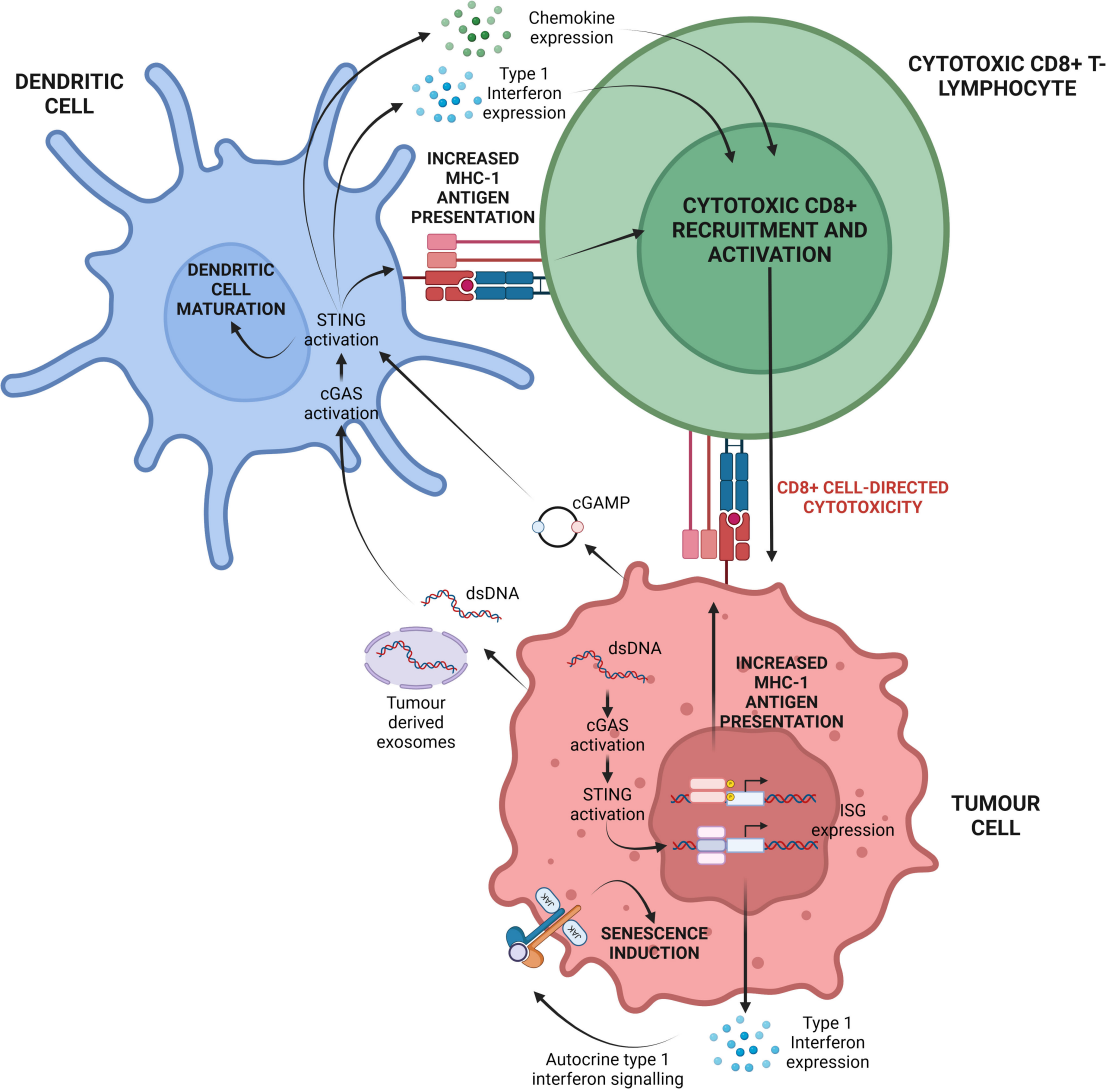
Depiction of the central cGAS/STING paradigm in the TME. Cellular stressors, namely chromosomal instability and replicative stress can result in the release of dsDNA into the cytosol. cGAS may subsequently recognise this dsDNA and induce cGAMP synthesis. Ultimately this may activate tumour-cell intrinsic cGAS/STING signalling and ISG expression. Alternatively, tumour cell stress can lead to the release of dsDNA, mtDNA and cGAMP into the extracellular space. These molecules can be internalised by dendritic cells present in the TME leading to tumour-cell extrinsic cGAS/STING activation. Cumulatively, both forms of cGAS/STING activation potentiate CD8+ cytotoxic T-lymphocyte recruitment to, and activation within, the TME for enhanced tumour cell elimination. Created with BioRender.com. Adapted from (19).

Emerging research highlights the cGAS/STING pathway as a key immunomodulatory signal in various cancers. cGAS/STING activation occurs when damage-associated molecular patterns (DAMPs) are detected in the tumour microenvironment (TME), often induced by chemotherapy or radiotherapy. This activation, though not the therapy’s direct target, results from therapy-induced immunogenic cell death, which releases tumour DNA and mitochondrial DNA, triggering the STING pathway in surrounding immune cells. In genomically unstable cancers (i.e. cancers with a high degree of chromosomal instability), dsDNA released via exosomes can also activate cGAS/STING in immune and tumour cells (**Figure 2**) (35). This signalling plays a vital role in tumour cell apoptosis, immune cell recruitment, and enhanced antigen presentation. Type 1 interferons, such as IFN-α, further amplify this response by promoting CD8+ T-lymphocyte activity and suppressing regulatory T cells, thereby reducing immunosuppressive cytokines like IL-10 and TGF-β (20, 25, 36).

Additionally, cGAS/STING signalling influences the polarization of tumour-associated macrophages (TAMs), shifting them from an immunosuppressive M2-like phenotype to a pro-inflammatory M1-like phenotype. This shift enhances tumour cell elimination by promoting phagocytosis, tumour antigen presentation, and the production of pro-inflammatory cytokines and chemokines, such as TNF-α, CXCL9, CXCL10, and IL-1 (37–41).

### 3.2 cGAS/STING signalling as a modulator of angiogenesis

Beyond its impact on immune cell function, cGAS/STING signalling also affects the vasculature of the TME, influencing neo-angiogenesis, vascular integrity, metastasis, and immune cell infiltration, primarily mediated by Interferon Beta (IFN-β) signalling (25, 42, 43). In a murine melanoma model, stromal cells were identified as an integral source of type 1 interferons, especially IFN-β (42). Briefly, IFN-β displayed anti-angiogenic activity in this model and intratumoural administration of a STING agonist resulted in type 1 interferon-dependent upregulation of various genes in surrounding endothelial cells. Notably, the cell adhesion molecules Melanoma Cell Adhesion Molecule (MCAM) and cadherin-5 were upregulated, both of which were associated with CD8+ T-lymphocyte infiltration of the TME (42). Data also indicates that IFN-β may downregulate VEGF expression, thus suppressing neo-angiogenesis. An association has also been drawn between cGAS/STING signalling and restoration of vascular integrity, contingent on type 1 interferon signalling. Vascular normalisation in this context potentiated chemotaxis and transendothelial migration of immune cells with a simultaneous mitigation of tumour cell metastasis (43).

### 3.3 Tumorigenic effects of cGAS/STING signalling

While the cGAS/STING pathway shows promise as a complementary therapeutic target in cancer treatment, it plays a dual role in both tumour elimination and tumorigenesis. This paradox may be due to sustained low-level activation of cGAS/STING, which maintains chronic pro-tumour inflammation (19, 44). For instance high chromosomal instability has been linked to prolonged cGAS/STING activation (25, 45, 46). While persistent cGAS/STING activation promotes chronic inflammation, a hallmark of cancer (19, 47). Chronic inflammation in the TME is also associated with increased invasion, epithelial-to-mesenchymal transition (EMT), and metastatic potential (48). Furthermore, this type of cGAS/STING activation is linked to chronic NF-κB activation, driven by chromosomal instability-induced tumour cell-intrinsic micronuclei containing dsDNA (46).

**Figure 3:**
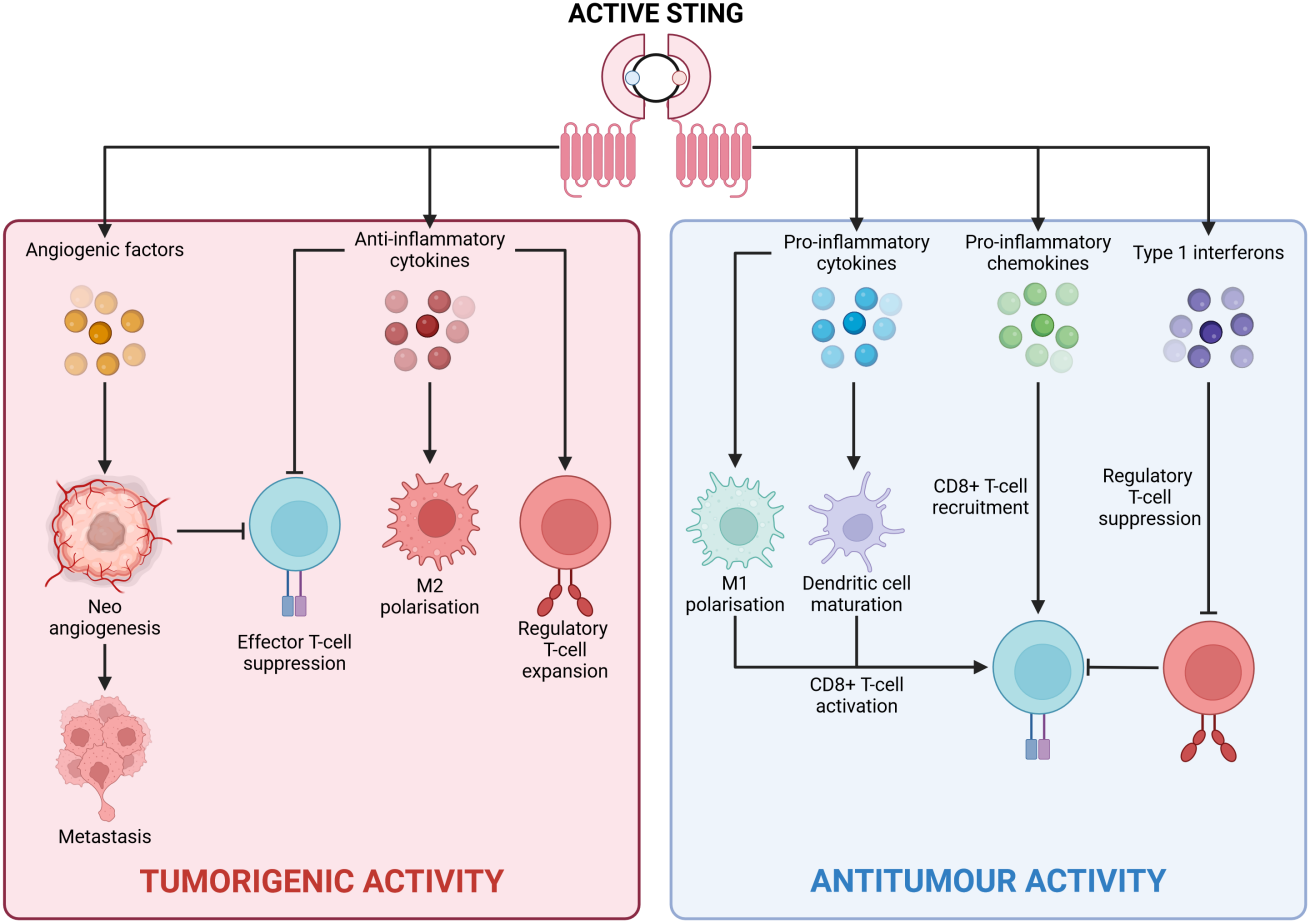
Divergent outcomes of cGAS/STING signalling. Active STING can drive two antithetical transcriptional profiles in a context dependent fashion with clear repercussions for tumorigenesis. Central to an anti-tumour inflammatory profile is the production of pro-inflammatory cytokines (such as IL-6), pro-inflammatory chemokines (such as CXCL10), and type 1 interferons (IFN-α/IFN-β). These inflammatory mediators promote tumour cell elimination via CD8+ T-cell mediated cytotoxicity. Contrastingly, a tumorigenic anti-inflammatory profile is underpinned by the secretion of anti-inflammatory cytokines (namely IL-10 and TGF-β), and angiogenic factors (VEGF). These anti-inflammatory proteins hamper the activity of tumoricidal immune cells while simultaneously potentiating tumour cell metastasis. Created with BioRender.com.

The cGAS/STING pathway also contributes to tumorigenesis by altering the metabolic profile of the TME. A Lewis lung carcinoma (LLC) study found that STING signalling induced the expression of the immunosuppressive molecule IDO, leading to tryptophan depletion, which supports regulatory T-cells and causes effector T-cell exhaustion. Interestingly, STING ablation enhanced CD8+ T-lymphocyte infiltration, increased anti-tumour activity, reduced myeloid-derived suppressor cell infiltration, and decreased IL-10 production (49). Similarly, in tongue squamous cell carcinoma, STING activation increased IL-10, IDO, and CCL22 levels, promoting regulatory T-cell recruitment and an immunosuppressive TME (50) (**Figure 3**).

Finally, the cGAS/STING pathway also plays a role in promoting tumorigenesis through non-canonical STING signalling that primarily activates NF-κB rather than IRF3. Briefly, following nuclear DNA damage, STING can be activated independently of cGAS by an ATM/IFI16 complex, leading to NF-κB activation (51). NF-κB drives tumorigenic pathways such as cell survival, proliferation, and chronic inflammation, and is linked to osteosarcoma pathogenesis (52, 53). Inhibiting NF-κB reduces the proliferative capacity of osteosarcoma cells (53) and suppresses tumour growth and angiogenesis in mouse models (54). NF-κB signalling also contributes to chemoresistance, anti-apoptotic protein expression, and metastasis in osteosarcoma (19, 55–58). Given the variable expression of cGAS/STING components and high chromosomal instability in osteosarcoma, some tumour subpopulations may activate NF-κB through non-canonical STING pathways, driving malignant progression even after cytotoxic treatment. Thus, understanding which signalling pathway is activated is crucial when targeting the STING pathway.

## 4 Targeting the cGAS/STING pathway in osteosarcoma

### 4.1 Expression of the cGAS/STING pathway in osteosarcoma

However, despite its potential for tumorigenesis, the cGAS/STING pathway is being explored as a novel immunological target for osteosarcoma due to its vital anti-tumour inflammatory effects. A previous study found impaired STING expression in osteosarcoma cell lines U20S and Saos-2 (22). These cells, along with an immortalized human lung fibroblast cell line (HEL), were infected with a mutant HSV-1 virus lacking the ICP0 protein. While HEL cells showed STING activation, U20S and Saos-2 did not, even when treated with the STING ligand 2’3’-cGAMP. Western blot and PCR analyses confirmed low STING levels in osteosarcoma cells but normal levels in HEL cells. Restoring STING in osteosarcoma cells reduced viral replication and increased ISG15 and IL-6 expression, indicating intrinsic STING deficiency. Together, the data indicate an intrinsic deficiency of STING expression in these human osteosarcoma cell lines at both the protein and gene expression levels.

Withers et al., 2023 further investigated STING pathway components in osteosarcoma cell lines, confirming significant STING downregulation in U2OS and Saos-2 cells compared to human osteoblasts (23). Saos-LM6 and MG63 cells had similar STING levels to osteoblasts, but MG63 cells showed reduced cGAS. Following X-ray irradiation, STING-deficient Saos-2 cells exhibited poor chemokine expression (CCL5 and CXCL10), whereas Saos-LM6 and MG63 cells expressed CCL5, and U2OS and MG63 cells produced CXCL10. siRNA-mediated STING knockdown in MG63 cells reduced chemokine expression, mirroring Saos-2 results. This study highlights the need for STING signalling in chemokine expression following DNA damage and reveals variations in cGAS/STING components among osteosarcoma cell lines, which may impact patient responses to radiotherapy. Most research on STING expression in osteosarcoma is based on *in vitro* cell line studies. While these studies provide valuable insights, it is important to acknowledge their limited translatability. Recently, an analysis of approximately 18,000 patient tumour samples from 139 different types of tumours was conducted using tissue microarrays. Among the 29 OS samples analysed, only 48.2% showed positive STING expression. Of this 48.2%, more than half (27.6%) exhibited weak STING expression, with only 10.3% of samples having strong STING expression (59). This data suggests that STING expression in OS is relatively low and often weak; however, similar to cell line data, there is variability in STING expression between patients. Unfortunately, there is still very little data available regarding the expression of cGAS/STING pathway components in patient samples, and further analysis is vital to truly understand the role the STING pathway plays in OS development.

### 4.2 Activating STING: a combined therapeutic approach

Although the cGAS/STING pathway plays an essential role in inducing an anti-tumour inflammatory response, the current approach does not favour relying on cGAS/STING as a standalone immunotherapeutic target. Instead, cGAS/STING activation is being used as a complement to other therapeutic approaches, specifically chemotherapy and radiotherapy. Sodium-glucose cotransporter 2 (SGLT2) was found to be overexpressed in osteosarcoma patient derived samples at the protein level, as confirmed by western blot analysis and immunohistochemical staining, though not at the mRNA level (validated by RT-qPCR) (60). Administration of SGLT2 inhibitors as monotherapies suppressed tumour cell growth while simultaneously promoting CD4+ and CD8+ T-lymphocyte infiltration. Importantly, SGLT2 inhibitor administration induced STING upregulation, at both the protein and mRNA levels in a dose- and time-dependent fashion. This upregulation is believed to occur through SGLT2 inhibitor-mediated suppression of PI3K/Akt signalling. Beyond STING upregulation, SGLT2 inhibitors also enhanced the phosphorylation of key pathway components, including STING, TBK1, and IRF3. SGLT2 inhibitors also increased the expression of IFN-β, which is central to the activation of the infiltrating immune cells. Silencing of STING suppressed all such effects, thereby confirming STING as the primary mediator of these effects. Critically, co-administration of 2’3’-cGAMP alongside SGLT2 inhibitors enhanced all aforementioned effects *in vitro*. Lastly, using a syngeneic murine model, SGLT2 inhibitors in concert with 2’3’-cGAMP constrained osteosarcoma tumour growth and potentiated CD45+ CD4+/CD45+ CD8+ T-lymphocyte infiltration at tumour sites.

Another combinatorial approach targeted the DNA damage checkpoint protein ataxia telangiectasia mutated and Ras3 related (ATR), a major regulator of the G2/M cell cycle checkpoint (61). Detection of DNA damage by ATR is one means of ATR activation therefore leading to cell cycle arrest, thus preventing cells with DNA lesions from replicating. This investigation centred on the combination of ATR inhibitors (VE822/AZD6738) with radiotherapy *in vitro* as a means of activating the cGAS/STING pathway the elicit an IFN-based inflammatory response. Herein, the levels of phosphor-STAT1 (pSTAT1) served as a measure of cGAS/STING activation and type 1 interferon activation. STAT1 phosphorylation is a known effect of autocrine and paracrine type 1 interferon signalling through interferon receptors. Application of low level (5Gy) Ionizing radiation (IR) alone to U2OS cells yielded increased pSTAT1 6-days post treatment. The combination of IR with ATR inhibitors expedited STAT1 phosphorylation in U2OS, despite the low levels of STING expression reported in these cells. Interestingly, STING levels increased following combination treatment, concurrent with increased generation of micronuclei. The localisation of cGAS to generated micronuclei is believed to be the precipitating factor. Indeed, cGAS co-localisation with micronuclei was detected in distinct intracellular foci. Lastly, siRNA-mediated depletion of STING abolished type 1 interferon production and STAT1 phosphorylation following combination treatment. Taken together, this data presents DNA damage checkpoint inhibitors as enticing anti-cancer agents when used in concert with radiotherapy. The suppression of DNA repair and activation of replication stalling, in conjunction with the generation of DNA lesions, supports the production of micronuclei due to cancer cell replicative stress. Micronuclei are known potent activators of the cGAS/STING pathway, which drives type 1 interferon production, providing a rationale for leveraging existing DNA checkpoint inhibitors as a novel means of eliciting an immunological anti-tumour response in combination with low-dose radiotherapy.

### 4.3 Activating STING: leveraging nanotechnological approaches

**Figure 4:**
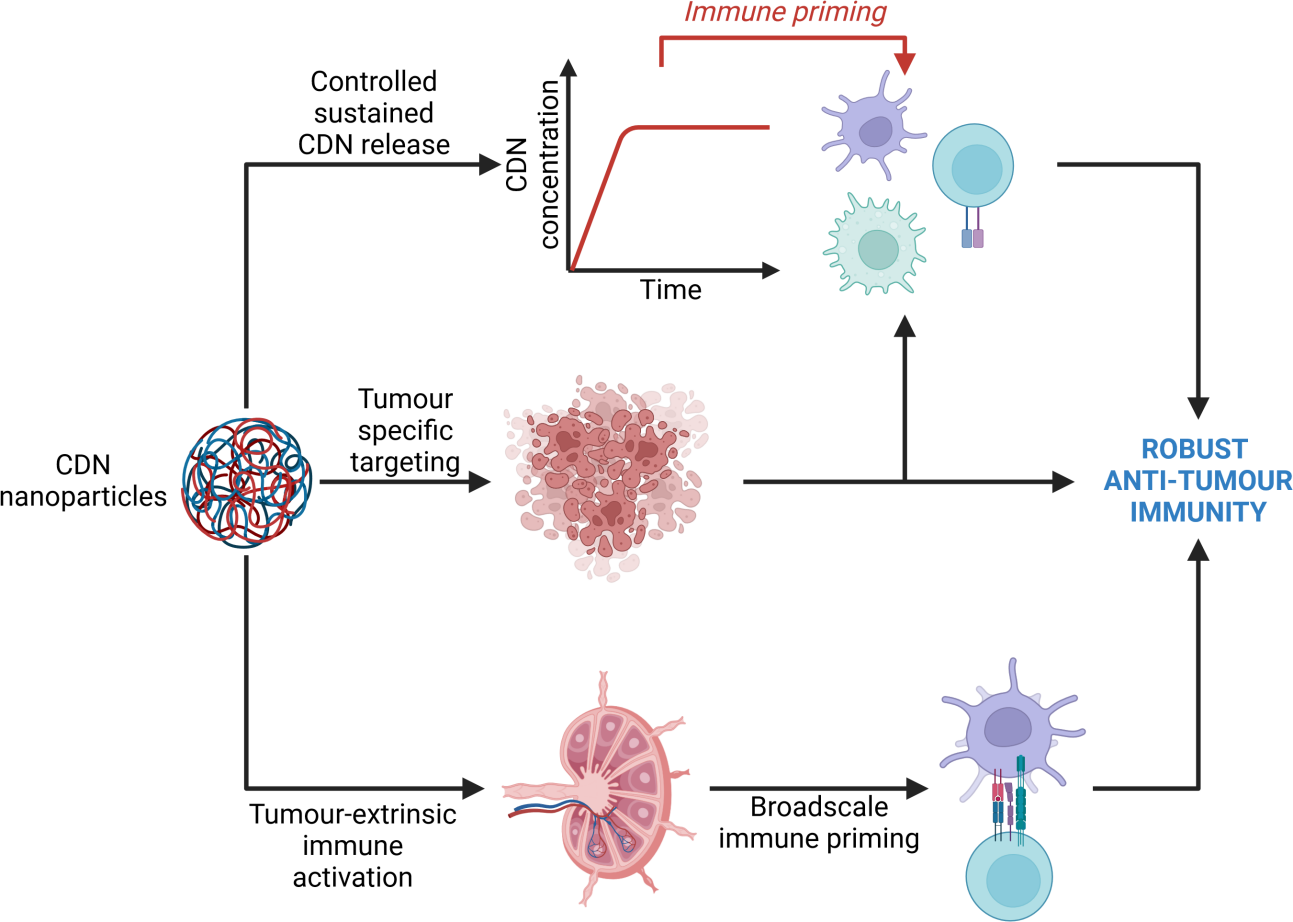
Avenues of immune activation by cyclic dinucleotide nanoparticles. Cyclic dinucleotide (CDN) nanoparticles primarily elicit an anti-tumour immune response through 3 main avenues. Firstly, nanoparticles may specifically enter the tumour microenvironment and support a tumour-intrinsic immune response by bolstering the activity of tumour-intrinsic immune cells. Secondly, nanoparticles may enter lymphoid organs wherein they are internalised and degraded by resident immune cells, namely dendritic cells, thus providing a priming signal to the immune system which may evoke a more potent anti-tumour immune response, while also supporting the elimination of circulating and metastatic tumour cells. Lastly, sustained release of CDNs from the nanoparticles supports sustained immune priming. Collectively, these three mechanisms of action may drive a more robust anti-tumour immune response. Created with BioRender.com.

The efficient delivery of the immunotherapeutic target continues to pose a significant challenge due to poor serum stability and cellular internalisation. Recently, innovative approaches using combinatorial strategies facilitated by nanotechnological drug delivery platforms, have been employed to activate cGAS/STING signal transduction. Nanotechnological delivery systems (NTS) are a popular contemporary research avenue for the treatment of various cancers. An NTS refers to a method for delivering drugs, genes, or other therapeutic agents to specific cells or tissues in the body using various nanoscale technologies. These systems include, but are not limited to, nanoparticles, nanosheets, nanofibers, nanocapsules. nanoframeworks, and nanocomposites. NTS’s allow the simultaneous employment of different therapeutic approaches simultaneously to achieve maximal therapeutic benefit. Depending on the formulation of the nanomaterials, small molecule drugs can be delivered alongside atypical therapeutic strategies such as photothermal therapy and photosensitization. The most common NTS are nanoparticle delivery systems (NDS). NDS have key advantages over typical treatment approaches which facilitate more innovative therapeutic approaches. Firstly, many chemotherapeutic drugs have broadscale toxicity with a range of off-target effects due to their lack of specificity. NDS can help reduce off-target toxicity by providing tissue- or site-specific delivery. This ensures that cytotoxic payloads are more precisely released at the site of action rather than systemically. Due to reduced toxicity, NDS also facilitate the administration of combination therapies that might otherwise have too high a toxicity profile. Lastly, NDS allow the contemporaneous delivery of different types of molecules, such as nucleic acids and adjuvants molecules like a STING agonist, along with chemotherapeutic agents to bolster anti-tumour effects of chemotherapy (**Figure 4**). A summary of these studies can be found in **Table 1**.

**Table 1:** Nanotechnological approaches which activate cGAS/STING signalling.

Model(s) utilised	Therapeutic formulation	Therapeutic target(s)	Results	Reference
HOS, 143BN and K7M2 spheroids K7M2 orthotopic mouse model	Pt(IV) prodrug encapsulated within an aldendronate-coated L-diisocyonate mPEG nanoparticle	Induction of DNA damage through Pt(IV) intracellular liberation	Nanoparticles displayed more potent anticancer activity versus cisplatin STING activation resulted in inflammatory cytokine expression and BMDC maturation Murine models displayed reduced tumour volume, M1 macrophage polarisation, and CD8+ T-cell infiltration	Shen et al., 2023 (65)
HOS, 143B, K7M2, and MG-63 cell lines K7M2-LUC orthotopic mouse model	Pt(IV) C-12 prodrug alongside NGL919 within an ROS-sensitive thiol-ketal encapsulation	NGL919 inhibits indolamine-2,3-dioxygenase to prevent tryptophan depletion Pt(IV) C-12 induces DNA damage following intracellular liberation	Nanoparticles Displayed more potent anticancer activity versus cisplatin Induced STING, TBK1, and IRF3 phosphorylation *in vitro* Induced dendritic cell maturation, CD8+ T-cell recruitment, M1 macrophage polarisation, reduced Treg populations	Xiang et al., 2023 (63)
LM8 cell line Bone marrow-derived dendritic cells LM8 orthotopic mouse model	Pegylated titanium carbide MXene nanosheets conjugated to an ovalbumin-Mn2+ ion complex	Nanosheets mediate NIR irradiation-induced cell cytotoxicity Liberated ovalbumin facilitates mt-DNA antigen presentation Liberated Mn2+ activates cGAS	NIR irradiation Induced LM8 apoptosis Induced Mn2+ liberation to drive dendritic cell maturation, cytokine expression, and cGAS/STING activation Demonstrated anti-cancer activity *in vivo* alongside increased inflammatory cytokine levels and CD8+ T-cell infiltration	Liu et al., 2022 (62)
K7M2 and U2OS cell lines K7M2 unilateral tumour mouse model	Tantalum-zirconium ligated to a photosensitising linker molecule to form TZM nanoparticles	X-ray irradiation induces the production of ROS	TZM nanoparticles Induced ROS generation and immunogenic cell death *in vitro* TZM nanoparticles in combination with X-ray irradiation and adjuvant anti-PD-L1 therapy Reduced local and distant tumour volume Induced dendritic cell maturation, inflammatory cytokine expression, and cGAS/STING activation	Li et al., 2023 (64)

For example, Liu et al.*, 20*22 developed PEGylated titanium carbide MXene nanosheets to which an ovalbumin-Mn2+ complex was bound (collectively referred to as TPOM nanocomplexes) (62). Ovalbumin served as an adjuvant antigen to support strong activation of dendritic cell (DC) maturation while Mn2+ ions were incorporated due to their notable enhancement of cGAS sensitivity for dsDNA, and the increased affinity of STING for cGAMP. The cell cytotoxicity of the TPOM nanocomplexes was first assessed by incubating TPOM nanosheets with LM8 murine osteosarcoma cells. In the absence of 808nm near-infrared (NIR) laser application, TPOM nanocomplexes were deemed biocompatible as no detectable death of LM8 cells was observed. In contrast, 808nm NIR laser administration to cells incubated with the TPOM complexes induced robust cell death in a time-dependent manner. Following confirmation of biosafety, 808nm NIR irradiation was shown to induce mt-DNA release within LM8 cells *in vitro*. Furthermore, using a transwell assay, 808nm NIR irradiation in combination with TPOM nanocomplex administration led to significant maturation of BMDCs due to the induction of tumour cell immunogenic cell death. Alongside mt-DNA and dsDNA release due to tumour cell irradiation-induced cell death, TPOM nanocomplex irradiation led to the liberation of ovalbumin and Mn2+ *in vitro*. Consequentially, the levels of BMDCs STING, phospho-IRF3, and IFN-β were elevated, revealing that TPOM nanocomplex irradiation is a biocompatible and potent means of activating BMDC STING signalling through the release of tumour antigens and co-stimulatory adjuvant molecules (ovalbumin and Mn2+). Lastly, *in vivo* murine models bearing subcutaneously implanted LM8 cell tumours were treated with a combination of NIR irradiation + TPOM and then compared to either NIR irradiation alone or no treatment. Ultimately, NIR irradiation of the TPOM nanocomplex led to efficient release of Mn2+ ions, ovalbumin, and mt-DNA synergistically activated dendritic cell cGAS/STING, culminating in improved dendritic cell maturation, enhanced tumour antigen presentation, increased inflammatory cytokine release, and increased CD8+ cytotoxic T-lymphocyte recruitment and activation at both local and metastatic tumour sites. These findings present this nanoplatform as a viable avenue for the synchronous activation of the innate and adaptive anti-tumour immune response through a multi-faceted stimulation of the cGAS/STING cascade. Importantly, a limitation to this study would be the subcutaneous injection of the LM8 tumour cells for the development of tumours. NIR irradiation does not possess the necessary penetration depth for use in an orthotopic model and therefore a degree of translatability is lost due to the non-native locations of primary tumours.

Another study synthesised a therapeutic composite nanoparticle containing both a platinum IV prodrug molecule (Pt(IV)-C12) and an indolamine-2,3-dioxygenase inhibitor (NGL919) (63). A critical feature of these nanoparticles was the presence of reactive oxygen species-sensitive amphiphilic thiol-ketal bonds. These polymers were used to encapsulate both molecules within the nanoparticles. The nanoparticles were observed to efficiently penetrate cancer cells after which the amphiphilic polymers would undergo reactive oxygen species-induced hydrolysis leading to intracellular release of Pt(IV)-C12 and NGL919. Pt(IV)-C12 induced DNA damage, subsequently activating the cGAS/STING pathway. NGL919 simultaneously inhibited indolamine-2,3-dioxgenase thus inhibiting tryptophan depletion in the TME. In response to nanoparticle administration, tumour cell apoptosis coincided with increased CD8+ T-lymphocyte recruitment and activation *in vivo* validated using a K7M2 (murine osteosarcoma cell line) orthotopic mouse model.

Tao Li et al.*, 20*22 developed a metallo-organic nanoparticle composed of a tantalum-zirconium framework combined with tetrakis(4-carboxyphenyl)porphyrin (TCCP) as a photosensitising ligand (64). *In vitro*, X-ray irradiation of nanoparticles induced extensive reactive oxygen species (ROS) generation. ROS generation, in conjunction with DNA damage caused by the X-ray itself, lead to immunogenic cell death of K7M2 and U2OS (human osteosarcoma cell line) cells that had internalised the nanoparticles. The apoptotic phenotype was deemed to be immunogenic based on the elevated levels of markers HMGB1 and calreticulin in the affected cells. Utilising murine models, intravenous injection of the TZM nanoparticles, accompanied by X-ray irradiation constrained to tumour sites, resulted in a significant reduction in tumour burden without detectable systemic toxicity. Next, TZM nanoparticles were co-administered with an anti-PD-L1 therapy followed by X-ray irradiation of tumour sites. This multifaceted approach led to profound immunogenic tumour cell death in both local and metastatic tumours. Tumour cell death was accompanied by the release of IFN-β, TNF-α, and IL-6 (attributed to cGAS/STING activation in the surrounding immune milieu following immunogenic cell death), all of which are drivers of anti-tumour inflammation. X-ray-induced DNA damage, coupled with the release of tumour cell nucleic acids following tumour cell death, efficiently activated cGAS/STING both *in vitro* and *in vivo*. Lastly, the release of tumour antigens, in conjunction with cGAS/STING activation, potentiated dendritic cell maturation and activation, thus facilitating the recruitment and activation of anti-tumour immune cells. Most recently, platinum prodrug-containing nanoparticles were synthesised via the incubation of Pt(IV) alongside L-lysine diisocyanate and mPEG (65). Following synthesis of the core nanoparticle, alendronate was conjugated to the nanoparticle exterior via electrostatic interactions. Essentially, nanoparticle composition capitalised on the high degree of glutathione overexpression in osteosarcoma tumours. Glutathione facilitated nanoparticle dissociation, thus releasing the cytotoxic phenanthridine molecule, derived from the Pt(IV) platinum prodrug. *In vitro*, fluorescence microscopy confirmed efficient internalisation of the nanoparticles in both murine osteosarcoma K7M2 2D cell monolayers and 3D spheroids. Intracellular concentrations of phenanthridine in these models were compared against unconjugated cisplatin as a measure of nanoparticle penetration and intracellular dissociation. Indeed, a 6-fold increase in intracellular phenanthridine was observed versus cisplatin alone, indicating efficient release and catabolism of Pt(IV) to active phenanthridine following cellular uptake. Next, researchers interrogated the capacity of their nanoparticles to induce DNA damage. Immunofluorescent staining of cytoplasmic dsDNA of K7M2 cells revealed significant DNA damage following nanoparticle uptake. Western blot analysis of cGAS/STING pathway components displayed a significant increase in STING, IRF3 and TBK1 phosphorylation following nanoparticle internalisation by K7M2 cells. The immunostimulatory effects of cGAS/STING activation in this context were assayed through co-culturing of K7M2 cells alongside immature bone marrow-derived dendritic cells (BMDCs). Importantly, BMDC maturation and secretion of type 1 interferons and IL-6 were enhanced following nanoparticle administration *in vitro*. Lastly, an orthotopic K7M2 murine osteosarcoma model was developed for the analysis of nanoparticle biodistribution and anti-tumour efficacy. Collectively, *in vivo* intracavitary administration of the alendronate coated nanoparticles displayed selective accumulation of the nanoparticles at tumour sites with minimal loss of Pt(IV) in circulation indicating excellent biosafety. Furthermore, alendronate nanoparticles yielded a 2-fold higher suppression of tumorigenesis versus cisplatin. Selective cytotoxicity of the nanoparticles was complemented by enhanced dendritic cell activation, increased CD8+ T-lymphocyte infiltration and activation, beneficial polarisation of tumour associated macrophages to an inflammatory M1-like phenotype, and elevated central memory T-cell levels in the spleens of treated mice. Researchers concluded that alendronate coating of the nanoparticles resulted in bone-specific accumulation and selective release of the active compound at tumour sites. This was coupled with efficient activation of the cGAS/STING pathway, through phenanthridine-induced DNA damage, resulting in type 1 interferon release and the genesis of an anti-tumour immune profile in the TME.

## 5 Future perspectives ad concluding remarks

Although targeting the cGAS/STING pathway seems to be a promising immunotherapeutic avenue, various limitations exist, resulting in a lack of clinical translatability to date. Firstly, many of the pre-clinical studies are conducted using murine models with agonists that effectively bind to murine STING (mSTING). Importantly, humans have 5 STING isoforms due to single nucleotide polymorphisms within the human STING gene, leading to significant differences in agonists binding between the different human STING isoforms (66). This has complicated the transition from murine models to human clinical applications due to discrepancies in agonist binding of STING and mSTING. Additionally, there is likely to be a collection of STING isoforms expressed within tumour cell subpopulations, where a single agonist is unlikely to sufficiently activate all isoforms. The chemistry of cyclic dinucleotides (CDNs) also has to be considered in both pre-clinical models and human therapeutics. Naturally occurring CDNs possess large molecules masses and tend to be strongly polar, these characteristics therefore impede their transmembrane transport and cellular uptake (67). Furthermore, natural CDNs are susceptible to hydrolysis by ectonucleotide pyrophosphatase/phosphodiesterase family member 1 (ENPP1) thus reducing the feasibility of systemic administration due to poor serum half-life (68). Interestingly, ENPP1 is reportedly upregulated in osteosarcoma therefore presenting a uniquely challenging environment for the administration of CDN-based therapies (69). Various synthetic CDNs have been developed to address this issue however, these synthetic analogues possess their own challenges. Synthetic analogues have improved cellular uptake and higher affinity for STING, which are beneficial traits. However, overactivation of STING signalling appears to impede the proliferation and survival of T-lymphocytes in a dose-dependent manner (67, 70). So, while CDN analogues have improved stability and affinity, they have a more restrictive therapeutic window, wherein immune activation has to be carefully balanced with the mitigation of toxicity (67).

The density and rigidity of osteosarcoma tumours will also likely impede the tumour perfusion of administered CDNs when administered via I.T. The other form of administration is through systemic delivery; however, this is hampered by the significant risk of off-target toxicity. STING is expressed across the body in various cell types. Therefore, the systemic administration of a STING agonist would likely lead to unsatisfactory immune responses outside of the tumour microenvironment (66). Lastly, osteosarcoma tumours often express high levels of immunosuppressive mediators, such as immune checkpoint proteins (PD-L1 and CTLA4), metabolic augmenters (indolamine-2’3-dioxygenase), and anti-inflammatory cytokines (such as IL-10) (71). These factors specifically reduce the feasibility of CDN monotherapies due to the high likelihood of insufficient and inconsistent STING activation.

With all of these limitations and obstacles to overcome, pertinent questions have been raised, including whether there is a therapeutic benefit to targeting the STING pathway for solid tumours like osteosarcoma, and if so, how we can effectively elicit a therapeutic response in such an immunosuppressive environment. At best, the generation of novel delivery systems like nanoparticle and hydrogel delivery systems will allow us to overcome the limitations of off-target toxicity and sustained release within the dense tumour microenvironment. At worst, the inflammatory response following STING activation may contribute to tumorigenic inflammation, namely via the NF-κB pathway, as previously described. This means that a strong yet constrained activation of STING in immune cells, particularly T-lymphocytes, needs to be achieved to prevent T-lymphocyte toxicity while simultaneously achieving tumour cell elimination. Therefore, for cGAS/STING stimulation to have a therapeutic response, it should be utilized as an adjuvant therapy to bolster and potentiate an anti-tumour innate immune response rather than as a target for monotherapy in osteosarcoma.

Cumulatively, it appears as though a combinatorial approach utilising chemotherapy and/or radiotherapy, alongside cGAS/STING agonists, leads to promising anti-tumour inflammation. Induction of cGAS/STING signalling seems to promote the recruitment and activation of dendritic cells and cytotoxic CD8+ T-lymphocytes, while simultaneously driving type 1 interferon and chemokine production. Results obtained both *in vitro* and *in vivo* indicate the potential of cGAS/STING induction as a promoter of TME anti-tumour immunosculpting. Monotherapeutic targeting of cGAS/STING has not been widely applied in osteosarcoma, instead, co-administration of 2’3’-cGAMP as a therapeutic adjuvant appears to yield optimal results. Careful consideration needs to be given to the degree and duration of cGAS/STING induction due to its previously described dichotomous role in tumorigenesis. Long-term activation of cGAS/STING may prove detrimental due to STING’s capacity to drive and perpetuate the formation of an intratumoural senescence associated secretory phenotype (72). Chronic inflammation, as a byproduct of SASP maintenance, is considered tumorigenic and detrimental to proper immune function (72, 73).Chronic inflammation, as a byproduct of SASP maintenance, is considered tumorigenic. Therefore, short-term potent activation of cGAS/STING is likely required to drive efficient tumour cell elimination while circumventing protracted STING activation. Tumour cell clearance, and the resolution of associated inflammation, likely requires a threshold of STING activation to occur. Investigating temporal regulation of cGAS/STING may be a promising avenue achieved through alternative sequences of therapeutic application alongside STING agonists. Nevertheless, cGAS/STING remains a promising novel immunomodulatory node with the potential to augment the immune composition of the TME across cancers. Leveraging cGAS/STING activation to bolster the action of existing chemo/radiotherapies is currently under intense investigation. We propose that delivering validated immunotherapeutics alongside agonists of cGAS/STING pathway components is a rational next step in the evolution of osteosarcoma treatment.

Promisingly, research leveraging biocompatible nanomaterial design and sophisticated nanoparticle delivery systems is being rigorously explored. The application of such technology for the contemporaneous administration of combination therapies has proven fruitful in the targeting of cGAS/STING in osteosarcoma. Many systems converge on the induction of DNA damage as a means of activating cGAS. The resulting production of type 1 interferons and inflammatory cytokines activates effective anti-tumour innate immunity. Nanoplatforms display enhanced biosafety versus their systemic counterparts, which is a valuable feature for the treatment of paediatric cancers, in particular. Systemic delivery of first-line chemotherapeutics exacerbates damage to the bone observed in osteosarcoma tumours. Therefore, any avenue that can mitigate deleterious side-effects associated with chemotherapies is invaluable in improving the quality of life of paediatric patients. Beyond the reduction of off-target toxicity, nanoplatforms display increased efficacy *in vitro* and *in vivo* with respect to tumour penetration, elimination, and engagement of immune effector cells. Current research also indicates the potential for nanoplatforms to enhance the anti-tumour effects of radiotherapy such as X-ray administration or near infrared photothermal therapy. Utilisation of nanoplatforms for the rapid and efficient activation of cGAS/STING at tumour sites may help us to circumvent the potential tumorigenic activities of cGAS/STING while still enabling us to benefit from the anti-tumour immunity elicited by cGAS/STING signalling.

In conclusion, significant progress has been made in investigating STING and its involvement in the immune-osteosarcoma domain. Nonetheless, numerous questions persist, particularly regarding the role of the cGAS/STING pathway in the interactions between osteosarcoma and the host microenvironment throughout various stages of treatment with proposed therapeutics such as radiation or chemotherapy. Once these aspects are clarified, leveraging cGAS/STING activation holds the potential to create a highly effective and indispensable combination treatment for osteosarcoma.
